# COVID-19 Vaccination and Disease Course in People with Multiple Sclerosis in Greece

**DOI:** 10.3390/jcm12175460

**Published:** 2023-08-23

**Authors:** Christos Bakirtzis, Natalia Konstantinidou, Sotiria Stavropoulou De Lorenzo, Theodoros Moysiadis, Marina-Kleopatra Boziki, Eleni Grigoriadou, Evangelia Kesidou, Paschalis Theotokis, Eleftherios Thireos, Panagiota Mitrou, Nikolaos Grigoriadis

**Affiliations:** 1 Multiple Sclerosis Center, Second Department of Neurology, School of Medicine, Aristotle University of Thessaloniki, 54621 Thessaloniki, Greece; nataliak95@gmail.com (N.K.); iradel7714@gmail.com (S.S.D.L.); bozikim@auth.gr (M.-K.B.); helengrigo@gmail.com (E.G.); kesidoue@auth.gr (E.K.); ptheotokis@auth.gr (P.T.); ngrigoriadis@auth.gr (N.G.); 2 Department of Computer Science, School of Sciences and Engineering, University of Nicosia, 2417 Nicosia, Cyprus; moysiadis.theodoros@gmail.com; 3 Primary Health Center of Vari, National Health System of Greece, 16672 Athens, Greece; ethireos@gmail.com; 4 Independent Department of Therapeutic Protocols and Patient Registers, Hellenic Ministry of Health, 10433 Athens, Greece; panagiotamitrou@gmail.com

**Keywords:** COVID-19, multiple sclerosis, vaccination, infection, SARS-CoV-2

## Abstract

Over the past three years, humanity faced the abrupt spread of COVID-19, responsible for a worldwide health crisis. Initially, it was believed that individuals with chronic disorders, including multiple sclerosis, were more likely to be infected and suffer a worse degree of COVID-19 disease. Therefore, data with regard to COVID-19 disease outcomes in these populations may provide additional insight with regard to the management of chronic diseases during viral pandemics. The objective of this study is to evaluate COVID-19 disease course in people with multiple sclerosis (PwMS) during the COVID-19 pandemic in Greece and explore the impact of vaccination in the outcome of SARS-CoV-2 infection in this population. Anonymized data, extracted from nationwide administrative records between February 2020 and December 2021, were retrospectively analyzed in order to identify PwMS with SARS-CoV-2 infection. Demographic data, as well as data regarding COVID-19 infection and vaccination, were additionally collected. The study sample included 2351 PwMS (65.1% females, 51.2% unvaccinated at the time of infection). A total of 260 PwMS were hospitalized, while 25 PwMS died from COVID-19 disease and its complications. Older age, male sex and the presence of comorbidities were independently associated with a higher probability of hospitalization. The risk of hospitalization was decreased in PwMS receiving some disease-modifying treatments. Anti-CD20s demonstrated high odds ratios without reaching statistical significance. Regarding fatal outcome, only age reached statistical significance. Vaccination provided a significant protective effect against hospitalization but did not exhibit a statistically significant effect on mortality.

## 1. Introduction

In the past three years, the spread of the COVID-19 pandemic has posed one of the greatest challenges humanity has faced in modern times. Over 650 million confirmed cases, and over 6.5 million deaths, were attributed to the severe acute respiratory syndrome coronavirus 2 (SARS-CoV-2) [1]. The consequent health crisis that followed affected almost everyone. However, healthcare professionals were more attentive to people with chronic diseases including multiple sclerosis (MS).

Since MS is an immune-mediated disease potentially capable of altering one’s immunocompetence, carrying a well-established increased risk for infections [2], people with MS (PwMS) were initially considered to be more vulnerable to severe COVID-19 disease course [3]. Additionally, a significant number of PwMS require relatively frequent hospital admissions either due to relapses or to administer infusion therapies, which make them more susceptible to SARS-CoV-2. Furthermore, the rising number of PwMS over 55 years old, usually presenting with several comorbidities and considerable accumulation of disability, was another reason that could impose concerns during the pandemic, since these characteristics constitute risk factors associated with worse outcomes after COVID-19 infection in the general population [4,5]. However, discrepancies were observed between preliminary studies in the Chinese population, where no higher risk of severe COVID-19 disease course in PwMS was found [6], and several French and Spanish cohorts, which demonstrated that PwMS were more likely to have a worse outcome after infection with SARS-CoV-2 [7,8]. Additionally, data from a larger cohort from the United States, Canada, the United Kingdom [9] and a recent systematic review [10] demonstrated equal chances of poor outcomes in PwMS and the general population.

Clinical manifestations of COVID-19 disease among PwMS are similar to those found in the general population (fever, cough, fatigue, shortness of breath, headache, etc.) [10]. Older age, male sex, comorbidities including cardiovascular diseases, diabetes mellitus, obesity, chronic pulmonary disease, high Expanded Disability Status Scale (EDSS) score, anti-CD20 treatments and high doses of glucocorticosteroids all seem to be associated with increased severity of SARS-CoV-2 infection in PwMS, with the vast majority accounting for risk factors in the general population as well [3,11,12,13,14].

Another challenging point for physicians during the COVID-19 pandemic was the use of disease-modifying therapies (DMTs), since they are associated with a greater risk of viral infections due to their immunomodulatory and/or immunosuppressive properties [15]. However, most DMTs do not have a large and long-term effect on innate immunity and CD8+ T cells [16], which constitute indispensable mechanisms for the elimination of SARS-CoV-2 [17]. Since the robust immune response itself is responsible for the severity of COVID-19 disease [18], DMTs do not actually seem to contribute to a poor outcome after SARS-CoV-2 infection [19], with the exception of anti-CD20 therapies [14]. Moreover, the potentially protective role of some DMTs (interferon beta, fingolimod) was also further investigated in several studies [20,21,22]. Most associations of the neurological community have published clear guidance in favor of the use of DMTs during the pandemic in order to control MS disease activity and potentially protect PwMS from severe COVID-19 infection [23,24,25,26,27].

The approval of anti-SARS-CoV-2 vaccines, apart from hopes, has also raised questions about their effectiveness in PwMS under DMTs. In several studies, only PwMS under anti-CD20 and—to a lesser extent—fingolimod treatment presented a rather reduced humoral response to vaccination; for PwMS under other DMTs, the antibody levels were comparable to those of controls [28,29,30]. Regardless of the low humoral response, these PwMS seemed to obtain a protective cellular response after vaccination [31,32,33]. A COVID-19 vaccination timeline has been proposed in order to improve immunization of PwMS under different DMTs [34]. Regarding side effects, short-term SARS-CoV-2 vaccination reactions were comparable to those observed in the general population [35,36]. Nevertheless, over 20% of PwMS [37,38] hesitated to get vaccinated due to safety concerns. Although MS relapses or acute onset of demyelinating disease after COVID-19 vaccination have been reported [39,40,41,42], no increase in the rate of relapses is indicated by studies so far [35,36]. Undoubtedly, SARS-CoV-2 vaccination in PwMS is recommended by experts, since the risks of severe COVID-19 disease course outweigh the risks of vaccination [34].

According to the Greek National Public Health Organization, there were 945,095 confirmed cases of COVID-19 in Greece from the beginning of the pandemic (end February 2020) to 1st December 2021, with 50.6% of the patients being male. Until then, the total number of deaths was 18,234, 95.4% of whom were people with associated comorbidities and/or age ≥ 70 years [43,44]. Until the 1st of December 2021, the number of people who had received at least one vaccination dose was 7,079,093, the fully vaccinated people were 6,639,951 and the number of people who had received a booster shot was 1,509,419 [44,45]. The first confirmed case of the omicron variant in Greece was detected on the 2nd of December 2021, so most of these data correspond to the alpha and delta variants of SARS-CoV-2, which were associated with more severe clinical manifestations and an increased risk of death compared to the omicron variant [46].

The prevalence of MS in Greece, according to administrative records, is estimated to 188.9 per 100,000 people, accounting for about 21,000 PwMS [47]. In this study, we aimed to evaluate COVID-19 disease course in PwMS during the alpha and delta SARS-CoV-2 variant pandemics in Greece, using nationwide administrative records. We additionally explored the impact of vaccination in the outcome of SARS-CoV-2 infection in this population.

## 2. Materials and Methods

In this study, we retrospectively analyzed anonymized data derived from the Greek national digital prescription database, where the COVID-19 registry is also included. The study period extended from the 26th of February 2020 (first COVID-19 case identified in Greece) until the 1 December 2021, focusing on the period of the pandemic, mainly attributed to the alpha and delta strains of the virus. In order to identify PwMS with SARS-CoV-2 criteria, we applied the following criteria: (i) having a positive test for SARS-CoV-2 infection either via rapid antigen test (RAT) or polymerase chain reaction (PCR), (ii) prescription of MS-related treatments with the ICD-10 code of MS (G35) for a period of at least 6 months, (iii) at least two consecutive prescriptions using the ICD-10 code G35, (iv) prescriptions predating positive testing for COVID-19. Demographic data including the age, gender and data with regards to COVID-19 infection, such as hospitalization and death, were additionally collected. Data related to MS, such as the type of MS, the duration of the disease and the EDSS score, were not available. In the analysis, major comorbid conditions such as hypertension and other cardiovascular disorders, dyslipidemia, other autoimmune disorders, diabetes mellitus, chronic pulmonary disease, and malignancies, whose presence had already been associated with a higher risk of severe disease course, were also included [4,12].

The statistical analysis included standard descriptive statistics for quantitative (mean and standard deviation) and qualitative variables (frequency, percentage). Age, sex, DMTs and number of comorbidities were compared between the unvaccinated and vaccinated groups of the identified PwMS. Appropriate hypothesis testing was applied for specific comparisons (*t*-test, chi-squared test) related to hospitalization and death from COVID-19. The corresponding *p*-values were adjusted in each case using the Bonferroni correction. To evaluate the impact of the independent factors (age, sex, DMTs, number of comorbidities and vaccination status) on hospitalization, and, separately, on death from COVID-19, univariable and multivariable binary logistic regression analyses were applied. All DMTs were considered as distinct categories within the regression analysis and were compared to the category representing “none DMT”. Regarding the number of comorbidities, the categories one, two, three and more than or equal to four comorbidities were treated as distinct categories within the regression analysis and were compared to the category representing no comorbidities. Regarding the vaccination status, the categories partial, full and boost were merged into one category reflecting vaccination, and this, as a whole, was compared to the unvaccinated category. The level of significance was set at 0.05 in all cases of hypothesis testing. The statistical analyses were conducted using SPSS v27 and the R programming language, v4.2.1.

This study was performed in accordance with the Declaration of Helsinki and its later amendments, in compliance with the national legislation on data protection (32.1289/24-04-2019) and received ethical approval from the local institutional ethical committee (7.381/7/20.04.21). Informed consent of the study participants was waived by the ethical committee.

## 3. Results

According to the statistical analysis, a total of 2351 PwMS (1531 or 65.1% females) tested positive for SARS-CoV-2 infection during this period, either using PCR methods (1351 or 57.4%) or with the use of a RAT (1000 or 42.5%). Out of these infected PwMS, 1204 (or 51.2%) were not vaccinated at the time of SARS-CoV-2 infection, while all other PwMS were partially (*n*: 84) or fully vaccinated with (*n*: 234) or without a booster dose (*n*: 829). Vaccination was performed with BNT162b2 (*n*: 920), ChAdOx1-S (*n*: 99), mRNA-1273 (*n*: 86) and Ad26.COV2-S (*n*: 42). Regarding the immunomodulatory treatment, 288 (12.2%) PwMS were not being treated with any DMT, whereas 1213 (51.5%) were receiving oral DMT administration, 717 (30.4%) were treated with injectables and 133 (5.6%) were treated with monoclonal antibodies at the time of the SARS-CoV-2 infection. The most commonly recorded comorbid conditions with particular interest for this study were dyslipidemia (*n*: 279 or 11.8%), hypertension (*n*: 245 or 10.4%), other cardiovascular diseases (*n*: 131 or 5.5%), chronic pulmonary diseases (*n*: 92 or 3.9%) and diabetes (*n*: 83 or 3.5%). Demographic data and data about DMTs and the number of comorbid conditions in the study population are presented in Table 1.

**Figure 1:**
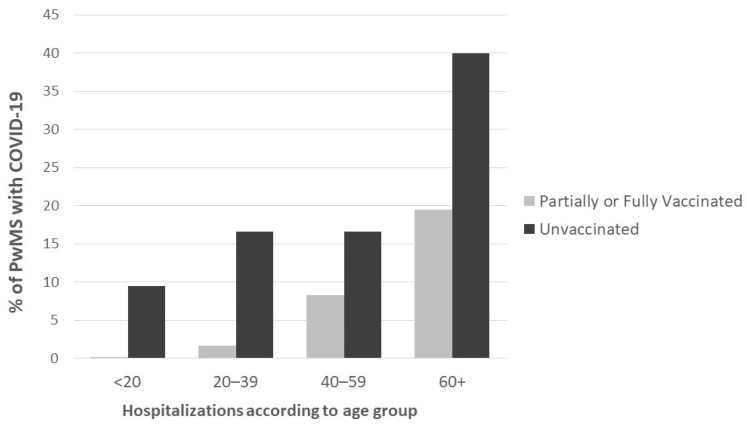
Hospitalizations according to age group. PwMS: people with multiple sclerosis.

With regard to the COVID-19 disease course, 260 PwMS (116 or 44.6% males, *n*: 79 or 30.4% partially or fully vaccinated) were hospitalized while 25 PwMS (*n*: 13 or 52.0% males, *n*: 8 or 32.0% partially of fully vaccinated) died due to COVID-19 disease and its complications. Among the study participants, those who were hospitalized due to SARS-CoV-2 infection were older (53.2 ± 11.6 vs. 42.2 ± 12.1, 95% CI: (9.4–12.4), *p* < 0.001), and presented more comorbid diseases (0.8 ± 1.2 vs. 0.3 ± 0.7, 95% CI: (0.4–0.6), *p* < 0.001). Hospitalization was more often observed in males (116/820 or 14.1% vs. 144/1531 or 9.4%, 95% CI: (1.8–7.6), *p* = 0.002) and in PwMS that were SARS-CoV-2 infected without being previously vaccinated (181/1204 or 15.0% vs. 78/1147 or 6.8%, 95% CI: (5.7–10.8), *p* < 0.001). Hospitalizations stratified across various age groups are presented in Figure 1.

**Figure 2:**
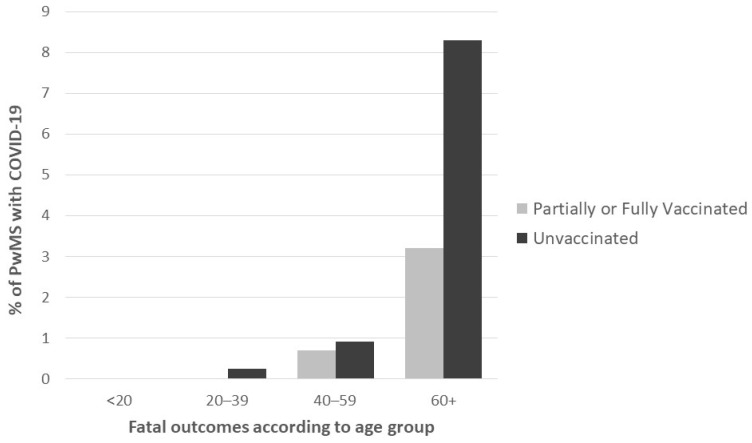
Fatal outcomes according to age groups and vaccination status.

Furthermore, those PwMS with a fatal outcome due to COVID-19 were older (61.0 ± 11.3 vs. 43.2 ± 12.4, 95% CI: (12.8–22.6), *p* < 0.001) and presented more comorbidities (1.3 ± 1.3 vs. 0.4 ± 0.8, 95% CI: (0.4–1.5), *p* = 0.006). Death was recorded in 1.6% of males and in 0.8% of females (95% CI: (−0.3–1.9), *p* = 0.444) and was more often observed in unvaccinated PwMS (17/1204 or 1.4% vs. 8/1147 or 0.7%, 95% CI: (−0.2–1.6), *p* = 0.548). Figure 2 presents fatal outcomes due to COVID-19 according to age group and vaccination status.

According to the univariable binary logistic regression analysis, the probability of hospitalization significantly increased with the presence of comorbid conditions, male sex and age, and decreased with partial or full vaccination and the presence of DMTs. Similarly, the probability for death due to COVID-19 was found significantly increased with the presence of comorbidities and age, and marginally increased with male sex. The presence of DMTs only marginally affected the outcome; however, some variations were observed within DMTs. Vaccination demonstrated a marginal tendency towards a protective effect with regard to death due to COVID-19 but did not reach the statistically significant level that was set. The results of the univariable analysis are presented in Table 2.

The relationship of the above factors with hospitalization and death due to COVID-19 was further examined with multivariable binary logistic regression analysis (Table 3). According to the results, male sex, age, the presence of comorbid conditions and the absence of SARS-CoV-2 vaccination were independent factors for increased rates of hospitalization due to COVID-19. DMTs seemed to provide a protective effect against hospitalization; however, it should be noted that, as expected, PwMS under treatment with DMTs were significantly younger than those without treatment with DMTs (mean age 42.1 ± 11.9 vs. 52.8 ± 12.4, *p* < 0.0001). With regard to death due to COVID-19, according to the multivariable analysis, only age was found to increase the probability of fatal outcome. The remaining factors did not exhibit statistical significance, perhaps due to the small number of PwMS who died due to COVID-19 during this time period.

## 4. Discussion

In this study, we aimed to explore the potential impact of age, sex, DMTs, comorbidities and vaccination in the course of COVID-19 disease in PwMS using administrative records from the national prescription database. According to the results, age, male sex and the presence of comorbidities were found to be the most significant poor prognostic factors for hospitalization.

With regard to male sex and age, previous studies with large study samples have provided similar results. In a recent analysis of 5648 PwMS from 27 countries, male sex, older age and higher disability rates were associated with worse COVID-19 disease course [48], while in a metanalysis of 30 relevant studies with PwMS, male sex was found to be an independent risk factor for a severe COVID-19 disease course [13]. It should be noted that male sex is also considered a risk factor for a poor COVID-19 outcome in the general population [49]. The presence of comorbidities has led to similar results in other registry-based studies [3,13] and has been associated with an increased risk of hospitalization and poor outcome in the general population as well [50].

According to this study, vaccination provided a significant protective effect against hospitalization. The effectiveness of vaccination against COVID-19 in the prevention of severe disease in PwMS has been previously observed in various studies [51,52], although certain DMTs seem to reduce the effectiveness of vaccines. Since immunization with SARS -CoV-2 vaccines has been proven to be safe in PwMS [53,54], and the development of severe disease may trigger inflammatory activity and clinical deterioration of their neurological symptoms [13,52,55,56], vaccination against COVID-19 disease is recommended [35,37,38]. The impact of vaccination on mortality risk did not seem to be significant in this study, perhaps due to the small sample size. Nevertheless, both studies in the general population [57] and in other neurological immune-mediated diseases [58] demonstrated the protective effect of these vaccines on hospitalization and mortality. Therefore, according to the updated Centers for Disease Control and Prevention (CDC) guidelines, people who are moderately or severely immunocompromised should receive these vaccines [59].

Initially, the outburst of COVID-19, followed by increased admissions in intensive care units (ICUs) and mortality rates worldwide, as well as the lack of understanding of the underlying pathophysiological mechanisms implicated in COVID-19 disease, alarmed healthcare professionals and posed serious questions regarding the safety of PwMS receiving DMTs. Already in March 2020, the Multiple Sclerosis International Federation addressed this matter, suggesting that despite the potentially increased risk of developing severe COVID-19 infection, the risks following DMT treatment discontinuation should also be taken into consideration, respectively [23]. Additionally, multiple MS societies and experts recommended the postponement of treatment initiation and treatment completion of DMTs associated with significantly high risk for infections as well as the discontinuation of treatment in case of confirmed SARS-CoV-2 infection [24,25,26]. A retrospective study revealed a significant decrease in the prescription of intravenously administrated DMTs as well as setbacks in treatment administrations [60]. However, further research on the underlying immune response triggered by SARS-CoV-2 infection and the effect of DMTs revealed relatively diverse results.

Both the innate and adaptive immune system play a key role in the immune response against SARS-CoV-2 [61]; however, the vast majority of DMTs target components of adaptive immunity, leaving innate immunity intact. Several studies were conducted on PwMS receiving DMTs, with some of them showing favorable results. Numerous studies revealed that PwMS using DMTs have an equal risk of infection to the general population [19]. Moreover, a cohort from Austria claimed that the risk of developing severe disease, as well as mortality rates, in patients treated with DMTs was equal to the general population [62]. Another cohort conducted in France supported this result by showing that there is no association between DMTs and severe disease [8]. Furthermore, several studies showed that the use of DMTs is associated with a lower risk of hospitalization and DMTs may therefore improve the outcome of these patients [48,63]. In this study, the risk of hospitalization was decreased in patients receiving some DMTs. This finding could be attributed to the immunosuppressive properties of these agents, which may prevent the development of COVID-19-related complications such as multisystem inflammatory syndrome [27] and may therefore reduce readmission rates [22].

Anti-CD20 DMTs are monoclonal antibodies targeting the adaptive immune system, particularly B cells, causing depletion [64]. B-cells are able to produce immunoglobulin M (IgM) and G (IgG), which serve as neutralizing antibodies directed specifically at a certain virus, preventing both primary infection and reinfection [16,61]. Ocrelizumab has the longest-lasting effect on B-cells amongst other anti-CD20 monoclonal antibodies [65]. Apart from B-cell depletion, anti-CD20 treatments exhibit a relatively slight effect on CD8+ and CD4+ T-cells expressing the CD20+ molecule. In particular, ocrelizumab shows steady 6–8% depletion of CD8 and 1–2% of CD4 T-cells and has a limited impact on monocytes [66]. This effect of anti-CD20 treatments—mainly on humoral, but also to some extent on cellular immunity—raised questions regarding the severity of COVID-19 infection and immunization after anti-SARS-CoV-2 vaccination in PwMS under these DMTs [67].

It has been demonstrated that humoral response after SARS-CoV-2 infection is reduced in PwMS under anti-CD20 therapies [62] and is influenced by treatment duration [68]. Several cases of prolonged COVID-19 disease course have been reported in patients receiving anti-CD20 monoclonal antibodies for any reason [69,70,71,72,73,74]. This seems reasonable, since elevated antibody titers have been detected in people with a shorter duration of SARS-CoV-2 RNA positivity [73]. However, according to Iannetta et al. [75], cellular responses after COVID-19 infection are present in PwMS under anti-CD20 treatment, although the extent of the response seems to be correlated with the amount of time between the infection and the last infusion.

In this study, in the multivariate analysis, anti-CD 20 therapies (ocrelizumab and rituximab) demonstrated high odds ratios, but with wide confidence intervals and without reaching statistical significance. The relevant sample size was relatively small (118/2351 or 5.0%) and there were not enough events in order to examine the effect of anti-CD20s on mortality. However, a notable number of studies have demonstrated a strong association between anti-CD20 therapies and severe COVID-19 disease. The analysis of the largest cohort on PwMS and concomitant COVID-19 infection [48] showed that the use of rituximab is associated with increased risk of hospitalization, ICU admission and need for artificial ventilation, whereas ocrelizumab administration is associated with increased risk of hospitalization and ICU admission. Sormani et al. [76] demonstrated an association between treatment duration and COVID-19 severity, while other researchers revealed an increased mortality risk in patients under anti-CD20 therapies who received the most recent dose closer to the time of COVID-19 infection [14,48]. A study performed on pediatric PwMS reproduced the aforementioned results, revealing an increased risk of infection, hospitalization and ICU admission in children receiving anti-CD20 therapy [77]. On the contrary, other studies concluded that anti -CD20s are not related to severe disease course [78], while intact B-cell populations are not a prerequisite for COVID-19 recovery [79]. Nevertheless, since anti-CD20s are often used in people with high disability levels and are linked to various infections, they should be used with caution on people with additional risk factors, such as comorbidities and older age, during a viral pandemic [80].

As far as postvaccination immunization is concerned, various serological studies have shown that PwMS receiving anti-CD20 monoclonal antibodies present with lower antibody titers compared with the general population due to the impaired humoral response; however, cellular immunity response was sustained [28,29,30,31,32,33,81,82]. Longer amount of time since the last anti-CD20 treatment dose, small number of infusions prior to vaccination and immunization with an mRNA vaccine—preferably mRNA-1273 vaccines—constitute positive predictors for higher antibody titers after SARS-CoV-2 vaccination [29,30].

This study was performed with data collected from a nationwide prescription database. Valuable information regarding other risk factors for severe COVID-19 disease course, such as levels of disability, previous MS history including DMT switches, smoking habits and subject weight, was not available. Nevertheless, this study demonstrated the beneficial effects of vaccination and provided additional data to support that age, male sex and the presence of comorbid conditions are risk factors for a poor COVID-19 disease outcome.

## 5. Conclusions

According to the results of this study, the risk of hospitalization due to COVID-19 exhibited a significant increase in the presence of comorbidities as well as with male sex and older age in PwMS. Vaccination and treatment with some DMTs decreased the risk of hospitalization, showing a protective effect. The risk of death was increased in older PwMS, and marginally in males, whereas comorbidities presented increasing tendency for a fatal outcome. Although vaccination did not significantly decrease mortality risk, some protective effect was revealed. There was no association found between treatment with DMTs and a fatal outcome.

Overall, the presence of MS by itself does not seem to affect the risk of hospitalization or the outcome of the disease [83]. However, comorbid conditions pose several challenges in the management of PwMS that are SARS-CoV-2 infected; additional treatment agents for comorbid conditions often lead to polypharmacy [84] and may further implicate the disease’s management. Clinicians are suggested to closely monitor aged, SARS-CoV-2-infected PwMS with significant comorbid conditions. Additional modifiable risk factors, such as smoking status and vitamin D3 levels, should also be considered [85].

## Figures and Tables

**Table 1:** Descriptive statistics of the study sample.

	Unvaccinated (*n* = 1204)	Vaccinated (*n* = 1147)
Age (mean, SD)	44.0 (12.5)	42.9 (12.6)
Male sex	430 (35.7%)	390 (34%)
Disease-modifying treatment		
None	168 (14.0%)	120 (10.5%)
Glatiramer acetate	151 (12.5%)	124 (10.8%)
Interferons	237 (19.7%)	205 (17.9%)
Teriflunomide	86 (7.1%)	78 (6.8%)
Dimethyl fumarate	248 (20.6%)	238 (20.7%)
Fingolimod	202 (16.8%)	259 (22.6%)
Cladribine	34 (2.8%)	43 (3.7%)
Azathioprine	14 (1.2%)	11 (1.0%)
Alemtuzumab	2 (0.2%)	0 (0.0%)
Natalizumab	36 (3.0%)	26 (2.3%)
Ocrelizumab	21 (1.7%)	35 (3.1%)
Rituximab	5 (0.4%)	8 (0.7%)
Number of comorbidities		
0	913 (75.8%)	872 (76.0%)
1	174 (14.5%)	173 (15.1%)
2	65 (5.4%)	57 (5.0%)
3	36 (3.0%)	38 (3.3%)
4+	16 (1.3%)	7 (0.6%)

**Table 2:** Univariable binary logistic regression analysis for hospitalization and mortality.

Outcome	Independent Factors	Odds Ratio	95% C.I. for Odds Ratio	*p*-Value
Hospitalization				
	Age	1.076	1.064–1.089	<0.001
	Male sex	1.587	1.223–2.060	0.001
	DMT			<0.001
	ALEMTUZUMAB *	-	-	-
	AZATHIOPRINE *	0.455	0.152–1.365	0.160
	CLADRIBINE *	0.166	0.065–0.425	<0.001
	DMF *	0.197	0.129–0.300	<0.001
	FINGOLIMOD *	0.291	0.197–0.428	<0.001
	GLATIRAMER ACETATE *	0.208	0.125–0.344	<0.001
	INTERFERON *	0.143	0.089–0.231	<0.001
	NATALIZUMAB *	0.039	0.005–0.287	0.001
	OCRELIZUMAB *	0.584	0.288–1.183	0.135
	RITUXIMAB *	0.716	0.192–2.668	0.619
	TERIFLUNOMIDE *	0.276	0.157–0.455	<0.001
	Number of comorbidities			<0.001
	1 **	2.449	1.770–3.390	<0.001
	2 **	2.387	1.448–3.937	0.001
	3 **	6.989	4.240–11.522	<0.001
	4+ **	5.024	2.033–12.411	<0.001
	Partially or fully vaccinated ***	0.418	0.317–0.552	<0.001
Death due to COVID-19				
	Age	1.122	1.084–1.161	<0.001
	Male sex	2.039	0.926–4.490	0.077
	DMT			0.091
	ALEMTUZUMAB *	-	-	-
	AZATHIOPRINE *	-	-	-
	CLADRIBINE *	0.331	0.042–2.607	0.294
	DMF *	0.156	0.043–0.565	0.005
	FINGOLIMOD *	0.276	0.095–0.803	0.018
	GLATIRAMER ACETATE *	0.092	0.012–0.717	0.023
	INTERFERON *	0.114	0.025–0.520	0.005
	NATALIZUMAB *	-	-	-
	OCRELIZUMAB *	-	-	-
	RITUXIMAB *	-	-	-
	TERIFLUNOMIDE *	0.311	0.068–1.420	0.132
	Number of comorbidities			<0.001
	1 **	5.915	2.266–15.439	<0.001
	2 **	3.702	0.778–17.626	0.100
	3 **	12.693	3.733–43.155	<0.001
	4+ **	21.155	4.237–105.633	<0.001
	Partially or fully vaccinated ***	0.490	0.211–1.141	0.098

* Compared to the None DMT category. ** Compared to the zero number of comorbidities category. *** Compared to the unvaccinated category.

**Table 3:** Multivariable binary logistic regression analysis for hospitalization and mortality.

Outcome	Independent Factors	Odds Ratio	95% C.I. for Odds Ratio	*p*-Value
Hospitalization				
	Age	1.065	1.050–1.081	<0.001
	Male sex	1.639	1.231–2.184	0.001
	DMT			<0.001
	ALEMTUZUMAB *	-	-	-
	AZATHIOPRINE *	0.248	0.075–0.819	0.022
	CLADRIBINE *	0.442	0.165–1.186	0.105
	DMF *	0.423	0.266–0.673	<0.001
	FINGOLIMOD *	0.589	0.384–0.904	0.016
	GLATIRAMER ACETATE *	0.388	0.225–0.667	0.001
	INTERFERON *	0.231	0.139–0.383	<0.001
	NATALIZUMAB *	0.078	0.010–0.588	0.013
	OCRELIZUMAB *	1.471	0.675–3.207	0.332
	RITUXIMAB *	1.507	0.366–6.200	0.570
	TERIFLUNOMIDE *	0.391	0.215–0.711	0.002
	Number of comorbidities			0.007
	1 **	1.437	0.996–2.072	0.052
	2 **	0.790	0.444–1.404	0.422
	3 **	2.569	1.402–4.707	0.002
	4+ **	0.980	0.360–2.673	0.969
	Partially or fully vaccinated ***	0.380	0.281–0.513	<0.001
Death due to COVID-19				
	Age	1.110	1.061–1.162	<0.001
	Male sex	2.103	0.909–4.866	0.082
	DMT			0.944
	ALEMTUZUMAB *	-	-	-
	AZATHIOPRINE *	-	-	-
	CLADRIBINE *	2.026	0.226–18.155	0.528
	DMF *	0.648	0.158–2.657	0.547
	FINGOLIMOD *	0.964	0.294–3.165	0.952
	GLATIRAMER ACETATE *	0.235	0.028–1.984	0.183
	INTERFERON *	0.302	0.062–1.469	0.138
	NATALIZUMAB *	-	-	-
	OCRELIZUMAB *	-	-	-
	RITUXIMAB *	-	-	-
	TERIFLUNOMIDE *	0.684	0.138–3.391	0.642
	Number of comorbidities			0.255
	1 **	2.684	0.965–7.469	0.059
	2 **	0.705	0.124–4.024	0.695
	3 **	1.996	0.479–8.309	0.342
	4+ **	2.304	0.370–14.358	0.371
	Partially or fully vaccinated ***	0.488	0.201–1.185	0.113

* Compared to the None DMT category. ** Compared to the zero number of comorbidities category. *** Compared to the unvaccinated category.

## Data Availability

Raw data used in this study are property of the Greek Ministry of Health. Requests to access the datasets should be directed to https://www.moh.gov.gr/ (accessed on 20 January 2023).
